# A Meta-Analytic Framework for Developing Protocols to Attend Child and Adolescent Victims of Sexual Violence

**DOI:** 10.3390/ijerph19095233

**Published:** 2022-04-26

**Authors:** Flávia Fernandes Trevizan, Regiane Máximo Siqueira, Aílton de Souza Aragão, Hugo Henrique dos Santos, Fabiano Henrique de Oliveira Sabino

**Affiliations:** 1 Department of Production Engineering, São Paulo State University (UNESP), Av. Eng. Luiz Edmundo Carrijo Coube, 14-01, Bauru 17033-360, SP, Brazil; f.trevizan@unesp.br; 2 Department of Public Health, Federal University of Triangulo Mineiro (UFTM), Uberaba 38025-180, MG, Brazil; ailton.aragao@uftm.edu.br; 3 Department of Production Engineering, Unifafibe University Center, Bebedouro, São Paulo 14701-070, SP, Brazil; hugohs92@gmail.com; 4 Department of Nursing, Federal University of São Carlos, Sao Carlos 13565-905, SP, Brazil; fabianooliveira163@gmail.com

**Keywords:** violent behavior, sexual behavior, multi-criteria decision analysis, Delphi, DPSIR framework

## Abstract

Violence against children and adolescents is a global public health problem. In Brazil, there are challenging boundaries for professionals in the protection network in general and for health professionals in particular. Moreover, among other factors, there is the challenge of referral, due to weaknesses in decision making, given the nature of sexual violence and how it is managed by healthcare services. This study aims to propose a Meta-Analytic framework to support the referral of young victims of sexual violence, considering levels of severity, independent of factors such as how protection systems are structured and managed and the local laws in force. We propose a Meta-Analytic approach, developed using the fundamentals of Delphi and DPSIR (Drivers, Pressures, State, Impact, and Response Model of Intervention), from the perspective of Value-Focused Thinking. The Delphi method was structured in two stages: the first stage aimed to identify and classify typical cases of sexual violence; the second stage used the DPSIR model, with the aim of identifying the decision criteria for typical cases that occur in a given municipality. The main outcomes are: (i) the application of the modified Delphi participatory method within the context of local social policies; (ii) the construction of a value tree based on Value-Focused Thinking; and (iii) the identification and systematization of criteria that most interfere with the evaluation of cases of sexual violence, which can be used for multi-criteria decision making.

## 1. Introduction

According to the World Health Organization (WHO) Report [1], violence consists of the intentional use of physical force or even economic power that threatens or puts people or communities at risk of death, psychological suffering, underdevelopment, or deprivation of essential elements for the maintenance of life. The same report reveals the types of violence and their nature, among which is sexual violence, which influences physical, psychological, and social development [2] and can become an increased risk factor for self-inflicted violence [1].

It is likely that violence has always been part of the human experience [3,4], and violence against children and adolescents forms part of the Public Health agenda. For example, health professionals recognize the impacts of “shaken baby” syndrome on a child’s full development [3,4,5].

The Panorama of lethal and sexual violence against children and adolescents in Brazil, produced by UNICEF-Brasil [6] in partnership with the Brazilian Public Security Forum (FBSP), revealed that 34,918 intentional violent deaths occurred between 2016 and 2020—an average of 6970 deaths per year over this five-year period. In the case of adolescents aged 15 to 19 years, 31,000 deaths were recorded, and children aged 0 to 9 years accounted for 1070 deaths. The data from this study conclude that violence against this sector of the public is related to a variety of factors including gender, ethnicity, life cycle, region of residence, and the type of weapon used. Regarding children, 33% were girls, 44% were white, 40% were white, 46% died from the use of a firearm, and 28% from a white weapon or “physical aggression”. Among adolescents aged 10 to 19 years, 91% of victims were male; 80% were black; 13% died at home, and 83% of the deaths were due to the use of firearms. The same study showed that between 2017 and 2020 there were 179,277 recorded acts of sexual violence, being rape or rape of the vulnerable, in which the victims were between 10 and 19 years old. Children up to 10 years old accounted for 62,000 of the victims in this four-year period [6].

In 2020, a year in which the world was affected by the COVID-19 pandemic, there was a drop in sexual violence against children and adolescents in Brazil, according to UNICEF’s Panorama report [6]. However, a month-by-month analysis reveals an historical pattern. For example, although the months of March and May 2020, marked by social isolation measures, show a reduction in records held by protection agencies, the drop reveals a different story for analysts: an increase in cases that went unreported, rather than a reduction in them.

The origin and circumstance of violence is complex, since most events take place inside the home, away from the public eye [7]. The victims are mainly girls, ranging from new-born babies to 18-year-old adolescents, who are abused by the men who live with them on a day-to-day basis [8,9]. Instead of a relationship of trust, in which they depend on affection and support, they are abused through physical strength, threats, and blackmail. The literature, based on clinical data, shows that victims of sexual abuse suffer from numerous symptoms, including anxiety, nightmares, post-traumatic stress disorders, inappropriate sexual behavior, fear, school problems, depression, isolation, and suicidal behavior [10]. On the other side of the problem, Platt et al. [2] suggests that healthcare professionals faced with situations of sexual violence against children and adolescents may have feelings that hinder rational decision making, which in turn may affect the appropriate referral of a case.

Given the data presented in this study, one form of sexual violence that is prevalent in Brazil, and needs to be urgently addressed, is that of promoting sexual games involving children or adolescents, with the intention of stimulating or obtaining sexual satisfaction. Furthermore, the aggressors of such sexual violence are often of an older age and have greater psychosocial maturity than their victims [6] (p. 25); a relational inequality that thus indicates the intention to perpetrate violence [1].

The advanced stage of psychosocial development sets the tone for the abuse or sexual violence to which children and adolescents may be subjected. When they are subjected to sexual activity, they cannot understand the intricacies of the activity which takes place, as they have not developed sufficiently to consent to such activity or reflect on its consequences [2]. As far as Western society has advanced in its institutional protection systems, in the form of laws and policies, sexual violence and abuse challenge the values of protection for this vast and diverse demographic segment, since this form of violence is […] responsible for short-, medium-, and long-term physical, emotional, and social consequences [7] (p. 301).

Faced with the paradoxes that surround the relational contexts that promote violence, actions to promote the doctrine of integral protection need to be adopted, as opposed to episodic emergency protection, which is disconnected from other actions; these are situations that perpetuate underreporting and reveal the need for training programs for professionals in the Unified Health System (SUS) [11,12]. Overcoming this state of affairs requires the provision of a safety net: support that must welcome people and lead them to those services instituted for the purpose of protection through intersectoral public policies, as evidenced by the Brazilian Federal Constitution (BFC), Article 227.

The concept of a protection network in this study can be understood as the articulation of people and organizations, whether public or private, who share causes and projects, governed by the horizontality of relationships in a democratic and sympathetic way. Such a network must be guided by cooperation, connectivity, division, and a sharing of responsibilities and competences. Thus, it is a complex work methodology, one which aims to re-encounter the totality of processes as a way of countering the historical fragmentation/sectorization of public policy management [13,14,15].

Due to this ambiguity, projects dealing with sexual violence must be structured as a protection network, attending to patients, welcoming them in and then referring them, as proposed in Article 227 of the BFC, which established the Integral Protection Doctrine in line with UNICEF guidelines, in the early 1980s and brought it into effect through the implementation of public policies.

From this perspective, the present study aims to contribute to the work of the professionals of the institutions that receive children and adolescents as victims of sexual violence, regardless of the region, culture, and laws involved, such as the Sexual and Reproductive Rights of children and adolescents, as stated in the Statute of Children and Adolescents (1990), from the perspective of a Meta-Analytic framework. Furthermore, this study aims to support the effective action of professionals from institutions that, guided by the National Policy for the Reduction of Morbidity and Mortality from Accidents and Violence of 2001, welcome the many victims of sexual violence.

Being an interpersonal act, sexual violence is marked by taboos in many countries around the world. Brazil is no exception. One of these taboos is the “pact of silence” [15,16,17,18,19]. This pact or code is seen as a real barrier to protective action, as its roots are variously attributed to poverty, emotional dependence and incest [7], or the constant threats suffered by the victims of abusers, whether directly or indirectly [14].

### Context and Goals

Given the context shown, this study aims to answer the following research question: “What is the referral strategy that maximizes the well-being of a child or adolescent who has suffered sexual abuse, considering its severity, guaranteeing sexual and reproductive rights, and accompanying the case over time?” An approach to sexual violence from a Meta-Analytic perspective is fruitful, as it can be reproduced in different contexts if, independent of prevalent laws, it values and enhances the experiences of the professionals involved in the case. Thus, the methodological strategy shown has been proven to be adaptable to different contexts and scenarios of a protection network, since it does not depend on issues such as the structural and managerial development of a protection system or on local precepts. A case study of this methodology was successfully developed in a protection network in the municipality of Bauru, located in the central-west region of the state of São Paulo, Brazil.

The decisions proposed by the Meta-Analytic strategy are based on the seriousness of the sexual violence suffered. Although all cases of sexual violence are serious, the strategy used is not about making a value judgment but about recognizing the biopsychosocial demands of a victim and promoting assertive referrals to the services available in a given protection network. In other words, the effectiveness of the right to health and social protection, in the Brazilian case, depends on how effective longitudinal referral and monitoring are. It is from this coherent decision-making that revictimization, a new cycle of violence, can be avoided.

In the words of José Ricardo de C. M. Ayres [20], based on what was expounded by Heidegger [21] when referring to the act of caring as a decision-making process:

“to act “in-function-of” […] is to tend to a position largely determined by a situation that precedes the moment of decision, but which is restructured for and by the subject of the decision from the moment when, together with the other, this subject updates his existential project in the decision made” [21] (p. 72).

In these contexts, the present study aims to provide health professionals aid, through the application of a Meta-Analytic framework for tracking and reporting cases of the sexual abuse of children and adolescents. This framework includes disciplines from different sectors and provides a quick response to Human Rights guidelines and the implementation of the Statute of Children and Adolescents (1990). This can be achieved by adopting a methodology to support multi-criteria decision making. Since gathering data on and analyzing cases of sexual violence can be complex, a common denominator is sought, so that the victims can be seen and referrals made with greater coherence agreed between the social actors of a protection network.

The theme of violence, as a public health issue, combined with operational research methodologies, has been addressed in recent research, demonstrating the applicability of tools that favor greater assertiveness in dealing with violence in childhood. Monitoring victims, even when still young, will reduce cases of revictimization [22]. Lane, Munro, and Husemann [23] applied a set of methodologies to support organizational analysis in child protection systems in England. Santos, Souza, and Aragão [24] used the Analytic Network Process (ANP) method to develop referral flows for child victims of violence in a study conducted in Brazil. However, it appears that such studies require a detailed analysis of the alternatives for the reception/monitoring by the services available in a given location for victims of sexual violence. Furthermore, studies involving operational research methods to deal with this significant public health problem do not present integrated decision support methodologies.

The Meta-Analytic framework was developed based on decision support strategies with multiple criteria. Particularly, the application of the method called Measuring Attractiveness by a Categorical Based Evaluation Technique (MACBETH), developed in the 1990s by Carlos A. Bana e Costa and JC Vansnick, has been shown to be suitable for problems such as the one being studied, given its sociotechnical nature, that is, it allows complex problems to be structured and, also, since it is a constructive decision-making method, favors the interaction and participation of experts/professionals while structuring and analyzing a problem [25]. The MACBETH multi-criteria decision method stands out as an approach that requires qualitative judgments, expressed by experts, on the differences in attractiveness of peer-to-peer alternatives [25]. These judgments, which are entered into a Decision Support System called M-MACBETH, provide the decision maker, or group of decision makers, with a quantification of the relative attractiveness of the options.

Given the complexity of the cases handled, a multi-criteria methodology was combined with the Delphi participatory method and Value-Focused Thinking. The model was strengthened in accordance with recent recommendations in the literature, which emphasize the importance of using integrated methods, especially in complex problems [26].

The methodological proposal is presented below (Section 2), followed by the case study and application of the MACBETH method (Section 3). The results obtained are presented in Section 4, and, finally, the conclusions given in Section 5.

## 2. Materials and Methods

Marttunen, Lienert, and Belton [27] point out that there has been greater attention in recent literature on the combination of the concepts of the Problem-Structuring Method (PSM) and the Multi-criteria Decision-Making model. The authors state that the most critical step for a multi-criteria analysis is problem modeling; and when decision elements are not easily found or defined in the literature, the problem-structuring approach appears to be a formal option to establish such structure.

A Meta-Analytic approach was developed to apply the modified Delphi, designed especially for this work, and the MACBETH methods used successfully in complex problems, characterized by employing multiple experts with training in different areas of knowledge (psychology, nursing, sociology, etc.). These methods fall into a constructivist approach, used as a way of structuring decision-making problems arising from interactions between the facilitator and the experts working in a given protection network. This constant interaction and instant feedback of the results from all stages of the project justify the use of the method in this study [28]. As mentioned before, a modified Delphi process was proposed to identify typical cases, identify criteria, and establish a consensus between experts in the decision-making process. The sector coordinator, who is involved at every stage of a Delphi process, was responsible for setting the number of experts chosen at a face-to-face meeting. All sectors involved in the protection network were invited to participate in the research project presentation.

**Figure 1:**
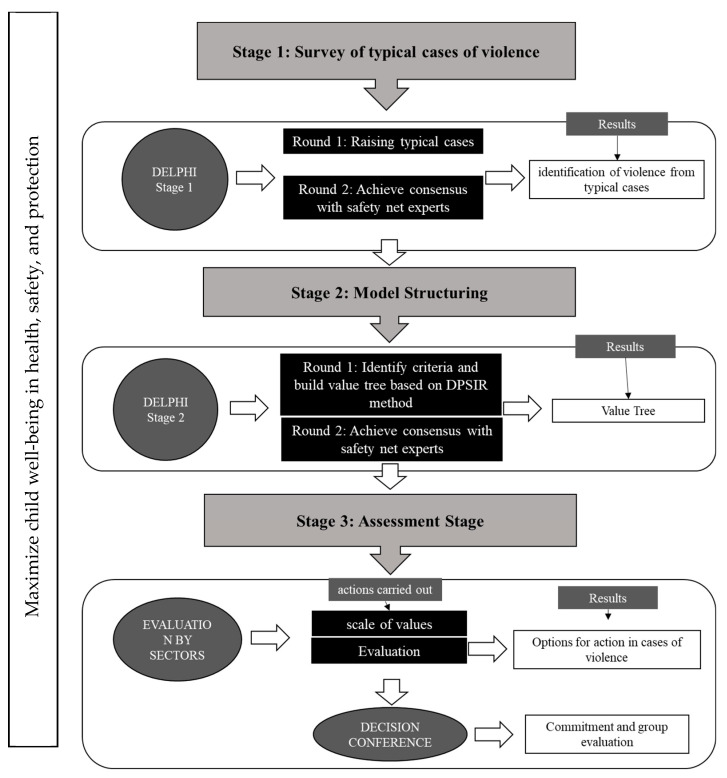
Methodological Framework.

Only decision makers in each sector participated in the research. Figure 1 shows the framework methodology of the study. It was divided into Stages, with absolute consensus and instant and interactive feedback of each playing an essential role in this regard. The steps are described below in more detail:

Stage 1. The modified Delphi process aims to identify typical cases from the experts working with sexual violence in a protection network, through statements made by those who experienced it. It then aims to identify criteria and build the value tree of the decision model. It is structured in two rounds.

In this first stage, the instructions given to responders on how to complete the first round of Delphi indicated that open-ended questions should be used to elicit as detailed answers as possible. The real names of those involved did not need to be identified at this stage. There was also a demographic form to complete, after which the research participant could: (i) see the previous answers, where the experts could access the aggregated answers of the research participants; (ii) edit the answers, which was possible to do until the end of the first round; and (iii) send another answer (i.e., be allowed to complete the form as many times as necessary). Before answering the questions, the professionals were given a description of the concept of sexual violence as defined by Priotto [29].

In the first round in this stage, the definition of sexual violence based on Magalhães [30] and ECA [31] was presented, and then three open questions followed: (1) Describe a case you have experienced that could be classified as sexual violence according to the definition presented. What was the referral performed (action), and what was expected to be achieved (objective) by this decision?; (2) What were your options for action in this case?; and (3) What were the determining factors for the success of the action? The responses were then placed in order and categorized by keywords. Only reports that had the same characteristics of violence were considered. After this, meetings were held with the Psychosocial-Child Monitoring Center (PCMC) and the Epidemiological Surveillance, Social Protection, and Child Protective Services—the healthcare institutions that make up the municipal protection network. Feedback from the results was presented during these meetings.

The second round was conducted by asking participants if they agreed with the results presented in the first round and if they would like to change their report or include something else. In this and at all stages, feedback is essential, and experts have the opportunity to review, add to, and agree or disagree with the results obtained in each round in which the Delphi method was applied. The idea was to analyze typical situations of sexual violence without the experts being subjected to an embarrassing situation or induced to remember a past personal experience, thereby ensuring that possible analysis biases were minimized and ethical precepts maintained.

Stage 2. In the second stage of the process, we presented one typical case identified from Stage 1. The first round for the present study was based on the integration of the Delphi method with the DPSIR Framework, which resulted in the following observations: Driving Forces: socioeconomic causes underlying the problems of sexual violence; Pressures: threats, coercion; States: changes caused to the individual by pressure; Impacts: the effect of sexual violence on the individual and the family; and Answers: the response of the protection network to the problem of sexual violence. In this round, further discussion was facilitated based on open questions:

(1) What are the possible socioeconomic causes underlying the problems related to sexual violence?
(2) What kind of oppression/threat can be provoked in an individual who has suffered sexual violence?
(3) What are the physical changes caused in an individual who has suffered sexual violence?
(4) What are the emotional changes caused in an individual who has suffered sexual violence?
(5) What are the psychological changes caused in an individual who has suffered sexual violence?
(6) What are the possible impacts on an individual and on their family caused by sexual violence?
(7) What are the possible responses that protection networks can offer to an individual who has suffered this type of sexual violence?

The results of the first round were supported by the set of keywords indicated and were statistically analyzed, which provided a basis for constructing the initial Value Tree presented at feedback meetings with the institutions, in the same way as in Stage 1. So, the second round was conducted during the feedback meetings with the experts from each sector, and the final structure of the Value Tree built with a very participative round where we obtained one hundred consensuses.

Finally, for each criterion considered in the analysis, a set of descriptors must be built. In order to establish the impact descriptors to evaluate each criterion, face-to-face meetings were carried out with representatives of each sector involved in the research. The descriptors proposed, based on Symptoms and/or Frequency and the levels of ‘good’ and ‘neutral’ impact were established.

We emphasized that sexual violence is not a “good” or even “neutral” event, as it is responsible for causing lifelong comorbidities, such as its impact on mental health [32]. Thus, this terminology does not evoke something desired or that does not justify an action, but it is coherent with the methods developed within the methodological framework of the present study: “neutral” is a threshold indicating that below it is an emergency case and above is an urgent case, and “good” is a threshold between an urgent case and an accomplishment case. The hope is for assertive decision making that prevents or reduces the revictimization of children and adolescents.

Stage 3. This stage is referred to as the evaluation phase of the MACBETH Model and it is divided into two parts: (i) an evaluation of the model criteria in each sector of the protection network, and (ii) a decision conference with all sectors involved in this research. In the first part, the evaluations in each sector were carried out through face-to-face meetings where each one analyzed the differences in attractiveness of the criteria, according to the specific case and to the MACBETH method. In the second part, a group evaluation was carried out through a decision conference. The decision conference aimed to present the results of all sectors involved, without identifying cases individually, to obtain a commitment to the results of the model and to analyze the impacts of applying the method in the context of the problem: sexual violence against children and adolescents. Figure 1 shows the methodological framework applied in this research.

## 3. Results

### 3.1. Case Study

The case study of the research was the Protection Network for children and adolescents, victims of violence, in the city of Bauru/SP, Brazil. Bauru is an average-sized city, with a population of 379,297 (2020). The Protection Network assists children and adolescents in situations of violence in different municipal sectors such as Health, Social Welfare, Education and Culture, Legal and Non-Governmental Organizations (NGOs), and Child Protection Services, which have administrative autonomy. The Child Protection Services, Health (Psycho-social Support Center—children (PSSC); Epidemiological Surveillance), and Social Protection sectors took part in this study. They all have a fundamental role in the attendance, welcome, follow up, and treatment of children or adolescents who have suffered some form of violation of their rights as citizens, as decreed by the 1990 Child and Adolescent Statute in Brazil [31]. The research was conducted between the years 2018 and 2019.

As described in Section 2, in order to form the panel of experts, a meeting was held with representatives of all sectors involved in the care of children and adolescents: representatives of the Department of Health, the Child Protection Services I and II, and the Department of Social Welfare (DSW) attended the meeting. The panel of experts comprised the coordinator of the sectors involved and professionals appointed by them, the latter of which varied according to their availability to take part in the meetings. All stages were conducted as described in Section 2.

#### 3.1.1. Stage 1: Definition of Typical Cases

In the first round of the modified Delphi method, which was to understand how a specific case that has already occurred is conducted, we obtained typical cases. The following is an example of a typical case obtained: “A case that I remember concerns a family in which three siblings were taken into care due to the physical violence they suffered from the stepfather and the mother’s omission. Prior to foster care, the children needed to be sent to emergency care, one of them being hospitalized and even undergoing surgery. After the reception, one of the siblings was diagnosed with syphilis. After contact with the Maternity Unit X, it was found that the child’s mother did not have this disease, and it was verified shortly after that the child had been sexually abused by his stepfather”.

To analyze the results, keywords were identified in all the reports, and, after counting how often they had been used, descriptive statistics were devised. As mentioned before, feedback played an important role at this stage, allowing the experts to interact with the results and include information and/or disagree with the keywords which had initially been defined. In all reports, violence had been committed by a family member (biological parents, stepparents, uncles/aunts, or grandparents). At this stage, characteristics of a typical case of sexual violence in children and adolescents in the region of Bauru/SP were defined by experts who participated in the study. It is important to emphasize that the statistics are drawn from each panel expert’s opinion based on their experiences. Table 1 shows the results.

#### 3.1.2. Stage 2: Structuring the Multi-Criteria Model

**Figure 2:**
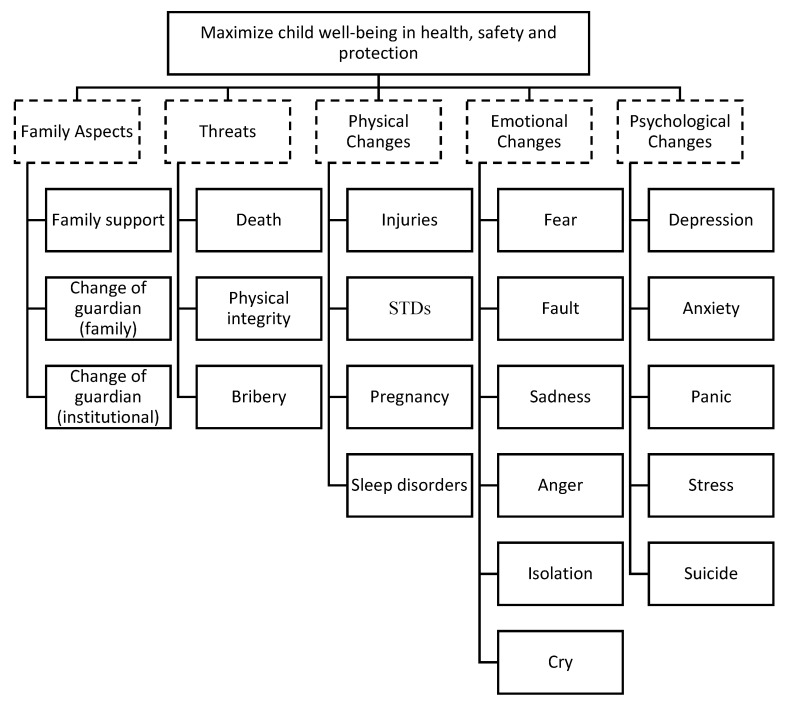
Keywords obtained.

The questions developed for this stage, using the DPSIR method, of the modified Delphi were established by observing the typical cases reported in the first round. The results were organized as keywords and organized in a tree map, as shown in Figure 2. At the first level, the objective of the tree map focused on values defined by the expert panel through the guarantees of health, safety, and protection. Afterwards, five areas of concern were defined: family aspects, threats, and physical, emotional, and psychological changes. The other factors were then allocated according to their association with one of the five areas of concern.

**Figure 3:**
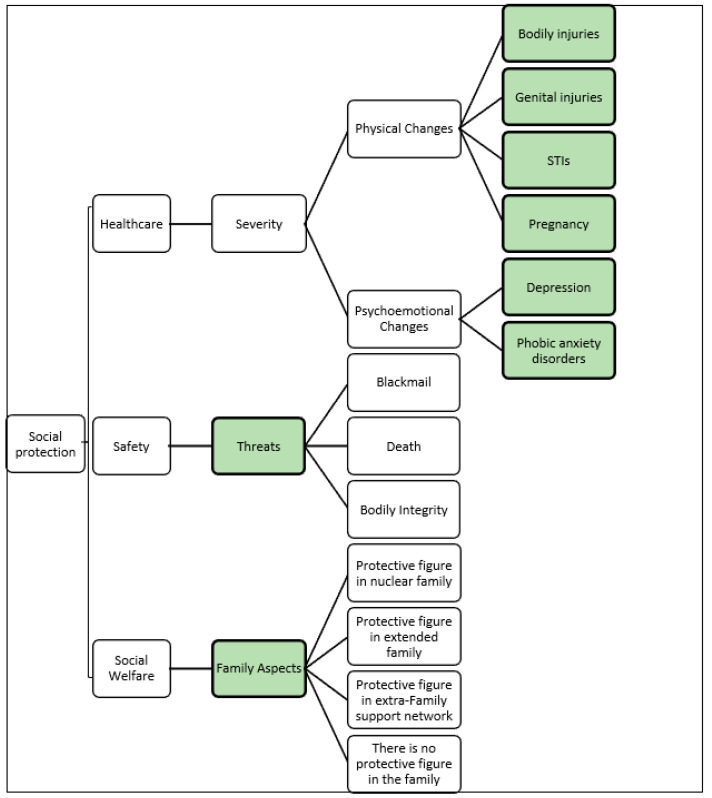
Value Tree.

At this stage of the process, during the feedback, Figure 2 was presented to the expert panel in face-to-face meetings and the second round, as described in Section 2, was conducted. The results were collected, and the criteria then structured with their experiences. The result is a Value Tree, which can be seen in Figure 3.

After constructing the Value Tree, the descriptors for each criterion were defined during face-to-face meetings designed for this purpose and held in each sector, during which a high level of interaction played an essential role. It shows how the professionals evaluate each criterion. Table 2, Table 3, Table 4 and Table 5 show the descriptors for each criterion based on the symptoms and/or frequency of the diagnoses, as well as the definitions of the ‘good’ and ‘neutral’ levels:

#### 3.1.3. Stage 3: Evaluation: Valeu Scales and Criteria Wights

As described in Section 2, this Stage was developed in two phases. In phase 1, four individual evaluation models were built for each participating sector. The swing waiting method was used to order the criteria according to importance and to build value scales for each criterion. Experts were required to give their opinion on the difference in attractiveness, through six categories: very weak (VW); weak (W); moderate (M); strong (ST); very strong (VST); and extreme (E) [27]. We collected the judgments of attractiveness through face-to-face meetings, for which a question protocol was developed based on the MACBETH methodology. Professionals were asked to assume that a child/adolescent had his/her state defined by neutral levels across the eight criteria. The experts were invited to discuss the following issue: “Suppose a child/adolescent meets all criteria at neutral levels, but the intention is to improve the child’s well-being. If you could improve one of the criteria levels, keeping everything else at the neutral level, which criterion would you choose?” This question was repeated until all the criteria were organized in decreasing order of attractiveness.

After making judgments about the difference in attractiveness between the criteria, the evaluation phase proceeded by obtaining information about inter-criteria. An example of a Bodily Injuries criterion question was: “In order to improve the health of a child/adolescent, moving from a state of serious injury to not injured, what is the attractive difference to the patient’s health?” In this work, improvements made to the worst level of performance were compared with the others and between each consecutive pair of performance levels, corresponding to the last column and the main diagonal of the decision matrix. The remaining questions within the criteria were answered through transitivity.

In phase 2 of this Stage, a decision conference was held to check the results and seek commitment to them in terms of the research. To model the opinions provided by the professionals, the following procedure was adopted: (1) As there were no individual judgment contradictions in the group model, all classifications of the individual models were considered; (2) With an inter-criterion factor, a decreasing classification was considered according to the criteria that appeared more than once in the same position; and (3) As there were no conflicting group result judgments, all classifications of the individual models were considered.

## 4. Discussion: Analysis of the Proposed Model and Value Scales

The results will be presented with comparisons among the sector, individual, and group models to explore the variability of opinions. For example, the criterion “bodily injuries” showed five levels of performance, from “no injury” to “very serious injury”, as shown in Table 6. Table 6 also shows the classification according to the MACBETH scale per Sector and the group view and the value scales of the individual and group models obtained via the qualitative judgments [23,24,25,26,27]. Light and Moderate levels are represented by the anchors “Good” and “Neutral”, so their scores are 100 and 0, respectively, in all situations.

Table 7 shows the classification of the criteria attributed by the sectors in the Protection Network. Gray cells show criteria that must be considered without distinction. For example, in the opinion of Sector I, bodily and genital injuries should be treated at the same time. In the opinion of Sector II, depression, phobic anxiety disorder, and bodily and genital injuries would be treated at the same time.

Table 7 shows that Sectors I and II considered the STI criterion as the most important, suggesting that this judgment can be interpreted by the severity of the diseases and the need to control symptoms and report them to healthcare services, as well as their severity for children, adolescents, and families.

**Figure 4:**
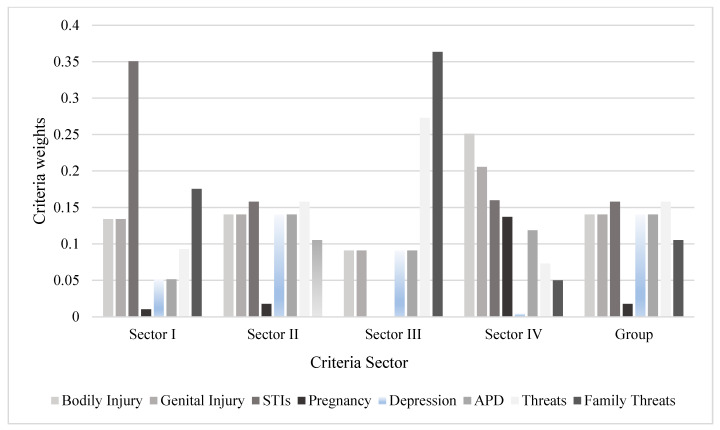
Criteria weights according to each Unit.

In light of the view from the group of professionals, faced with all the opinions provided in the Sectors, the classification obtained is given in decreasing order. Figure 4 shows the criteria weights based on the evaluation of each Sector and the Group. It was observed that the “STIs” criterion had the highest score for Units I (0.3505) and II (0.1578). The “Threat” criterion had the same score as the STI criterion in Unit II and the second highest score in Unit III (0.2728). “Depression”, “Phobic anxiety disorder”, “genital injuries”, and “bodily injuries” shared the same score in the view of the group (0.1404). The highest score found in all models was for “Family aspects” in Unit III (0.3636). Sector III did not feel comfortable answering questions about STIs and pregnancy because they understand that they are aspects related to health.

As the studies by Avanci, Pinto and Assis [33] and Rodrigues et al. [34] highlight, most acts of violence, especially physical (self-inflicted, beatings) and sexual (rapes and abuse) violence, involve healthcare services, especially emergency services, as highlighted by the WHO Report on Violence [1]. In addition, as recommended by the Statute of Children and Adolescents, in the Brazilian case, other sectors and their respective equipment will be activated, including Social Protection (Basic and/or Medium Complexity), Justice System (Public Ministry, Children’s Court), Education (Child or Elementary), Mental Health (Psychosocial Care Centers), and the Guardian Council itself, among others [35].

After evaluation of the MACBETH method phase, the typical case shown at the start of this section can be analyzed. The description of the case was characterized by severe bodily injuries, mild genital injuries, contamination by STIs, and no diagnosis of pregnancy. In this example, depression and phobic anxiety disorder were considered at the moderate level; the victim was threatened with death, and there was no protective family. With the evaluation of the case presented, global scores for the participants of each Unit and for the group as a whole could be obtained.

Table 8 shows the criteria organized according to the most negative, which means, using the MACBETH method, that these assessments are located below the “neutral” levels of the set of descriptors, establishing worse than satisfactory levels. Criteria with a score of 0 are located at the “neutral” level, those with a score of 100 are located at the “good” level, and those above 100 are considered levels that are better than “good”.

**Figure 5:**
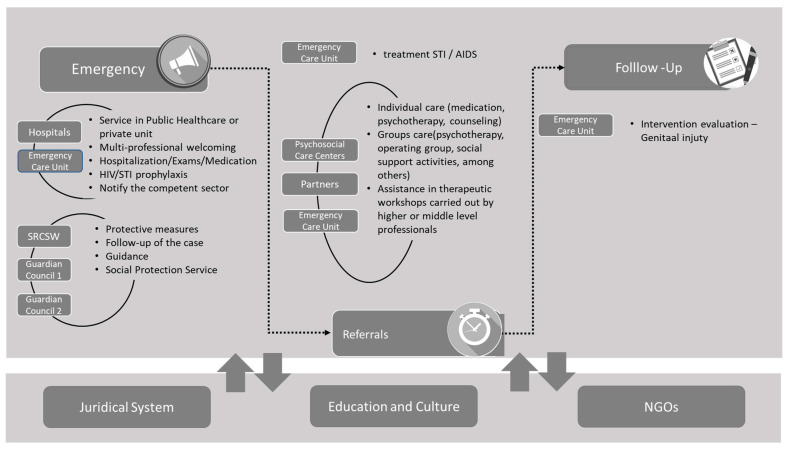
Options for the Protection Network to take, for the typical case available to participants based on the MACBETH method scores.

In the example given, to construct the individual referral of child and/or adolescent victims of violence, based on the model, we considered that a criterion requires urgent care (immediate treatment) by healthcare, social protection, and mental health services when it has a negative score. Zero score criteria are classified as referrals, i.e., treatments that can wait (do not require immediate intervention). Finally, criteria with a positive score must be followed up periodically. The view of the expert group came to the same conclusion, that is, there was no contradiction and/or divergence between answers. The diagram shown in Figure 5 illustrates the procedures described above, suggesting options for intervention for the urgent, referral, and follow up criteria. It is important to note that this method is dynamic and enables the evolution of each child’s treatment over time to be achieved.

It is important to emphasize that this structure is a suggestion that has arisen from the overview of representations of professionals, and that they are aligned with the Statute for Children and Adolescents (ECA) and with Brazilian guidelines and protocols, notably those published by the Ministry of Health and the National Council for the Rights of Children and Adolescents (CONANDA). Although professionals from the Justice Department, Education and Culture Systems, and NGOs were not included in the study, these institutional actors were constantly mentioned, highlighting their relevance to the structuring and functioning of the network with a view to comprehensive protection.

## 5. Conclusions

In view of the proposed objective, this study emphasizes that the meta-analytic approach allows for the provision of answers for decision making in a protection network, valorizing the experiences of specialists/professionals with different backgrounds, and providing an opportunity for the critical reflection of practices and regulations in their context. The most important result of this work is structured in a proposed framework, which can be adapted to different protection networks. The results show the importance of the treatment timeframe for the dynamic evolution of children, depending on the gravity of a specific situation. Furthermore, the Meta-Analytical approach can be implemented in any location, independent of culture and laws, because it begins with the experience of the professionals involved and the typical cases treated.

The method developed related the values and objectives prioritized by experienced professionals in the services of a given protection network to the states and severity of the SV cases they handle. The complexity of this form of violence demanded, the combination of multi-criteria methodology with the participatory Delphi and DPSIR methods using value-focused thinking. This strategy contributed to methodological improvement by using a Meta-Analytic approach, with the application of other approaches during research stages to verify the results. The method also contributed to decision making in the referral of children and adolescent victims of SV in the municipality, according to the severity of the demands.

Future studies could consider applying the same methodological strategy to other manifestations of violence in other locations/countries, the use of linear optimization tools to maximize the cost/benefit of measures per project to guarantee rights, and the development of free software to support the customized decisions of systems, such as those analyzed.

The methodology used demonstrated its potential in the prospection of integrated public health projects that rapidly connect emergency services, such as referral hospitals, emergency rooms, and referrals to other services in a protection network (Basic Health Units, Family Health Strategies; Child Psychosocial Care Centers) and their follow-up, with a view to reducing or even eliminating re-victimization and recidivism. The methodological strategy exercised here could be applied to different contexts, with different specialists in a Protection Network, and could focus on other types of violence, such as neglect and psychological and physical trauma.

This study contributes to the understanding of SV against children and adolescents from the perspective of professionals, as they identify the values that guide their action in the face of the objectives of the doctrine of integral protection in Brazil. Furthermore, developing or improving integrated health projects, supported by a logical process using mathematical tools, makes decision making consistent in the face of the persistent underreporting of this form of violence.

## Figures and Tables

**Table 1:** Statistics of typical cases of sexual violence: origin, referral, and objective reports.

Sexual Violence	
Source	Number of reports
Intrafamily	13
Case referral	Number of reports
Assistance at the guardianship council	7
Psychological support	6
Institutional care	5
Assistance in a medium-complexity social protection service	4
Opening a police report	3
Host in extended family	2
Hospital care	2
Legal Medical Institute Service	2
Public Prosecution Service and Judiciary Police Center	1
Removal of family custody	1
Goal	Number of reports
Full protection	11
Preservation of mental health	5
Strengthening of family bonds	3
Prevention or treatment of STIs	2
Ensuring legal protection	1
Prevention of risk/revictimization situations	1

**Table 2:** Descriptor for the “Bodily Injury” criterion.

Physical Changes		
Criterion	Symptoms and/or Frequency	Descriptors
Bodily injury	Symptoms Bruises Excoriation Burns Bites Blunt short injury Sizes Type of intervention	Lesions classified according to the type of intervention.
		Not injured Mild injury (requires outpatient treatment)—**Good level** Moderate injury (requires specialized treatment without hospitalization)—**Neutral level** Serious injury (requires hospital and/or specialized treatment with hospitalization)

Genital injury	Symptoms Bruises Lacerations Sizes Extent of injury	Injuries classified according to the AAST scale Not injured Bruise or hematoma—**Good level** Superficial laceration (mucous only)—**Neutral level** Deep laceration in fat or muscle Complex laceration, up to cervix or peritoneum Injury to adjacent organs (anus, rectum, urethra, bladder)
STIs	Sizes STI/AIDS diagnosis (yes or no)	There is a diagnosis of STIs or not There is no diagnosis—**Good level** There is diagnosis—**Neutral level**
Pregnancy	Sizes Pregnancy diagnosis (yes or no) Want to continue (prenatal or termination of pregnancy) Want to keep the child (mother keeps the child or give up baby for adoption)	Pregnancy diagnosis There is no pregnancy diagnosis—**Good level** There is a pregnancy diagnosis, she wants to continue and is interested in keeping the child—**Neutral level** There is a pregnancy diagnosis, she wants to progress, and there is no interest in keeping the baby There is pregnancy diagnosis, and she does not want to continue

**Table 3:** Descriptors for the “Depression” criterion.

Psychological Emotional Changes		
Criterion	Symptoms and/or Frequency	Descriptors
Depression	Symptoms Feeling depressed Guilt Suicide Initial Insomnia Intermediate insomnia Delayed insomnia Work and interests Delay Agitation Anxiety (psychic) Anxiety (somatic) Gastrointestinal total General total Genital Sizes Frequency Functionality (Degree of autonomy)	No depression Mild depression—**Good level** - Has some symptoms - Frequency: sometimes - Functionality: can maintain daily functions Moderate depression—**Neutral Level** - Has some symptoms - Frequency: frequently - Functionality: needs support to continue with daily functions and has family support Moderate depression with aggravating factor - Has some symptoms - Frequency: frequently - Functionality: needs support to continue with daily functions and does not have this support Severe depression - Has some symptoms - Frequency: always - Functionality: unable to continue with daily functions even with support Severe depression - Has some symptoms - Frequency: always - Functionality: life-threatening and/or attempts at suicide
Anxious phobic disorders	Symptoms Feeling Anxious Tense Fear Insomnia Intellectual Difficulties Feeling depressed Motor summation Sensory summation Cardiovascular symptoms Respiratory symptoms Gastrointestinal symptoms Genitourinary symptoms Neurovegetative symptoms Behavior in interview Sizes Frequency Functionality (Degree of autonomy)	No phobic anxiety disorder Mild disorder—**Good level** - Has some symptoms - Frequency: sometimes - Functionality: can maintain her daily functions Moderate disorder—**Neutral Level** - Has some symptoms - Frequency: frequently - Functionality: needs support to continue with daily functions and has this family support Moderate disorder with aggravating factor - Has some symptoms - Frequency: frequently - Functionality: needs support to continue with daily functions and does not have this support Severe disorder - Has some symptoms - Frequency: always - Functionality: unable to continue with daily functions even with support

**Table 4:** Descriptors for the “Threat” criterion.

Safety		
Criterion	Symptoms and/or Frequency	Descriptors
Threat	Size Blackmail Bodily integrity Death	Without any kind of threat—**Good level** With blackmail—**Neutral level** With threat to victim and/or third-party bodily integrity Threat of death to victim and/or third parties

**Table 5:** Descriptors for the “Family Aspect” criterion.

Safety		
Criterion	Symptoms and/or Frequency	Descriptors
Family aspect	Size Presence of a protective figure	Protective figure in nuclear family—**Good level** Protective figure in extended family—**Neutral level** Protective figure in extra-family support network (neighbor/ godparents) There is no protective figure in the family

**Table 6:** Value scales for “Bodily Injuries”.

Units	Bodily Injuries				
	There are No Injuries	Light	Moderate	Serious	Very Serious
Sector I	374.25	100.00	0.00	−100.00	−150.00
Sector II	180.00	100.00	0.00	−100.00	−400.00
Sector III	275.00	100.00	0.00	−25.00	−50.00
Sector IV	200.00	100.00	0.00	−50.00	−87.5
Mean	257.31	100.00	0.00	−68.75	−171.87
Stadev	88.03	0.00	0.00	37.5	157.57
Group	200	100.00	0.00	−25.00	−100.00

**Table 7:** Classification of Criteria for each unit.

Sectors	1st	2nd	3rd	4th	5th	6th	7th	8th
Sector I	STIs	Family aspects	Bodily injuries	Genital Injuries	Threats	Depression	Phobic anxiety disorder	Pregnancy
Sector II	STIs	Threats	Depression	Phobic anxiety disorder	Bodily injuries	Genital injuries	Family Aspects	Pregnancy
Sector III	Family aspects	Threats	Depression	Phobic anxiety disorder	Bodily injuries	Genital injuries	-	-
Sector IV	Bodily injuries	Genital Injuries	STIs	Pregnancy	Phobic anxiety disorder	Threats	Family Aspects	Depression
Group View	STIs	Threats	Depression	Phobic anxiety disorder	Bodily injuries	Genital injuries	Family Aspects	Pregnancy

**Table 8:** Evaluation by individual and follow up.

**Typical** **Case**		**Sector I**		**Sector II**		**Sector III**		**Sector IV**		**Group**	
		Criterion	Score	Criterion	Score	Criterion	Score	Criterion	Score	Criterion	Score
	1st	BI	−100	FA	−266.67	FA	−90	T	−200	FA	−266.67
	2nd	FA	−90.91	BI	−100	T	−50	FA	−166.67	T	−66.67
	3rd	T	−50	STIs	0	BI	−25	BI	−50	BI	−25
	4th	STIs	0	D	0	D	0	D	0	STIs	0
	5th	D	0	APD	0	APD	0	STIs	0	D	0
	6th	APD	0	T	0	GI	100	APD	0	APD	0
	7th	GI	100	GI	100.00			GI	100	GI	100

## Data Availability

The data presented in this study are available on request from the authors.
